# Understanding the Solvation Structure of Li-Ion Battery Electrolytes Using DFT-Based Computation and ^1^H NMR Spectroscopy

**DOI:** 10.1021/acs.jpcb.2c06415

**Published:** 2022-11-16

**Authors:** Julia Im, David M. Halat, Chao Fang, Darby T. Hickson, Rui Wang, Nitash P. Balsara, Jeffrey A. Reimer

**Affiliations:** † Department of Chemical and Biomolecular Engineering, University of California, Berkeley, Berkeley, California94720, United States; ‡ Materials Sciences Division and Joint Center for Energy Storage Research (JCESR), Lawrence Berkeley National Laboratory, Berkeley, California94720, United States

## Abstract

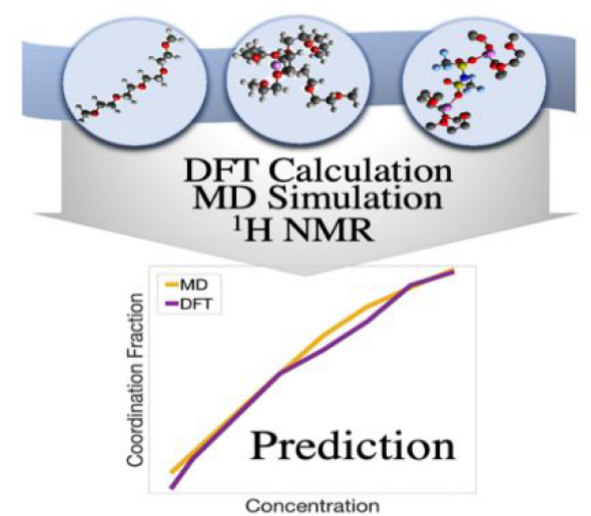

Molecular dynamics (MD) simulations, density functional theory (DFT) calculations, and ^1^H NMR spectroscopy were performed to gain a complementary understanding of the concentrated Li-ion electrolyte system, lithium bis(trifluoromethanesulfonyl)imide (Li[TFSI]) dissolved in tetraglyme. The computational methods provided the concentration dependence of differing solvation structure motifs by reference to changes in the corresponding NMR spectra. By combining both the computational and experimental methodologies, we show that the various solvation structures, dominated by the coordination between the tetraglyme (G4) solvent and lithium cation, directly influence the chemical shift separation of resonances in the ^1^H NMR spectra of the solvent. Thus, the ^1^H NMR spectra can be used to predict the fraction of tetraglyme involved in the solvation process, with quantitative agreement with solvation fraction predictions from MD simulation snapshots. Overall, our results demonstrate the reliability of a hybrid computational and experimental methodology to understand the solvation structure and hence transport mechanism of LiTFSI-G4 electrolytes in the low concentration region.

## Introduction

Ionic liquids (ILs) are low-melting-point solvents useful for a wide range of applications, including electrolytes in batteries, supercapacitors, and dye-sensitized solar cells.^1−7^ For example, solvate ionic liquids (SILs) represent one subclass of ionic liquids that comprise concentrated solutions of salt in a molecular solvent with high donor number. SILs are characterized by strong coordination of solvent to the dissolved ions, typically cations, to form stable, long-lived complexes. Such electrolytes are an example of IL utility for Li-ion battery applications because they contain only the working cation (Li^+^), as opposed to mixtures of Li-containing salts and traditional ionic liquids, which contain two types of cations and as a consequence may possess low Li^+^ transference.^8^ Glymes (i.e., methoxy-terminated oligoethers, CH_3_O(CH_2_CH_2_O)_*n*_CH_3_) are promising solvents commonly used as components of Li-ion battery electrolyte systems. Glyme-based systems, such as lithium bis(trifluoromethanesulfonyl)imide (Li[TFSI]) salt dissolved in tetraglyme (G4), have been extensively developed and explored.^1,9−12^ Recent work has shown this class of electrolytes undergoes greatly reduced decomposition in full battery cells compared to standard carbonate-based systems, enabling use of more experimental electrode materials and extending cycle life.^13^

Tetraglyme, which constitutes a typical molecular solvent of the SIL system, has also proven its potential in Li-ion batteries and supercapacitors; it is a small-molecule analogue of the quintessential poly(ethylene oxide) (PEO) material used as a solid-state polymer electrolyte, and its study can thus also provide insight into the ion transport mechanisms in polymer electrolytes.^14^ For example, typical characteristics of tetraglyme-based electrolytes as used in battery applications are high solubility of alkaline salts as well as high ionic conductivity.^14^

We surmise that developing more effective and more applicable battery electrolyte systems will rely on quantitative understanding of the mechanisms of ion solvation as revealed by local solvation structure,^14^ allowing for further fine-tuned control of the electrolyte properties.^15^ Various computational efforts have been undertaken to analyze the structure–property relationships in Li-ion battery electrolyte systems using methods such as molecular dynamics (MD) simulations and density functional theory (DFT) calculations.^1,15^ It has been mentioned previously that solely relying on computational methods to predict and analyze electrolytes, without recourse to experimental techniques such as spectroscopy, may lead to inaccurate conclusions.^15^

With the intention of providing further understanding of the LiTFSI-G4 electrolyte system, as well as to further complement the use of simulations as a computational tool for this system, we performed a series of MD simulations to understand the solvation structure and dynamics as a function of Li-ion salt concentration.^16^ Results from MD simulations were evaluated with DFT modeling to predict ^1^H NMR results, which were subsequently compared with experimental ^1^H NMR shifts, thereby supporting the specific solvation structures. Our results reveal the local solvation structure of glyme-based electrolyte systems in the low concentration regime (i.e., below 2.5 *m* or 2 M) by combining DFT calculations, MD simulations, and NMR spectroscopy. Furthermore, our results provide insight into the specific nature and dynamics of the interactions between the tetraglyme molecular solvent and the lithium cation.

## Methodology

### Density Functional Theory Calculations

DFT-based geometry optimization and NMR chemical shift prediction were conducted with the quantum chemistry Gaussian software.^17^ The basic molecular geometries were constructed using the auto-optimize tool of the Avogadro software^18^ using the Merck Molecular Force Field (MMFF94^19^) and Universal Force Field (UFF^20^). MMFF94 was the default force field used for optimizing tetraglyme, and UFF was used for structures containing Li^+^ and TFSI^–^ ions. The geometries were then optimized using the standard Gaussian method with the Becke, three-parameter, Lee–Yang–Parr (B3LYP) functional^21,22^ and using 6-31G(d) as the basis set. Following the calculations, frequency calculations were performed to confirm that the geometry-optimized conformation was at its energy minimum. NMR calculations were performed in Gaussian with the same level of theory and the same basis set as the geometry optimization calculations. Multiple functional and basis sets were compared, and the B3LYP/6-31G(d) combination was found to be sufficiently accurate (closely resembled the experimental results) with much greater time efficiency given the large number of structures considered.

### Sample Preparation and ^1^H NMR Spectroscopy

LiTFSI-G4 mixtures were prepared in a concentration range of 0.18–2.5 mol/kg of solvent. Herein the concentration of the samples is defined by *r* = [Li^+^]/[O], the ratio between the concentration of lithium cations and that of the oxygen atoms in tetraglyme, for consistency with previous work.^16^^1^H NMR spectra were acquired on the samples to identify the relative shifts of the resonances corresponding to protons within CH_2_ and CH_3_ groups of the tetraglyme solvent; additionally, ^1^H *T*_1_ (spin–spin relaxation) measurements were performed using a standard inversion recovery sequence. ^1^H NMR experiments were performed at a field strength of 16.4 T using a 700 MHz Bruker Avance I spectrometer, equipped with either a Bruker 5 mm double-resonance broadband observe (BBO) probe or a Bruker 5 mm triple-resonance inverse (TXI) probe, with variable-temperature control. Measurements were performed using a Larmor frequency of 700.1 MHz. The sample temperature was fixed at 30 °C.

### Molecular Dynamics Simulations

MD simulations were conducted on the LiTFSI-G4 electrolyte system. The selection of force fields for the LiTFSI salt and tetraglyme solvent as well as the simulation methods is similar to these reported previously.^16^ In brief, the Transferrable Potentials for Phase Equilibria with United Atom description (TraPPE-UA) force field is used for tetraglyme molecule.^23,24^ The compatible all-atom force field is used for LiTFSI salt.^25^ MD simulations are performed in the NPT ensemble (303 K, 1 bar) using the Gromacs code.^26^ The temperature is maintained using the velocity-rescale thermostat,^27^ while the pressure is kept using the Berendsen barostat.^28^ The bonds of tetraglyme molecules are constrained using the LINCS algorithm.^29^ The nonelectrostatic and electrostatic interactions are computed using the cutoff method (cutoff length: 1.2 nm) and the particle mesh Ewald (PME) method,^30^ respectively. The box size ranges from 3.32 to 3.66 nm. The number of tetraglyme molecules is set to 100, and the numbers of Li^+^ and TFSI^–^ ions vary in accordance with experimental salt concentration.

The residence time autocorrelation functions are defined to evaluate the time scales of the dominant solvation motifs.^31^ For a specific motif (see below), it is defined as *C*_motif_(*t*) = ⟨*P*_*m*_(*t*)·*P*_*m*_(0)⟩/⟨*P*_*m*_(0)·*P*_*m*_(0)⟩, where ⟨...⟩ denotes the ensemble average that is collected from all available motifs of the same type. *P*_*m*_(*t*) is a binary function, which is equal to 1 when an individual motif is always made of the same ions and G4 molecules over time *t* and is equal to 0 otherwise.

## Results and Discussion

**Figure 1:**
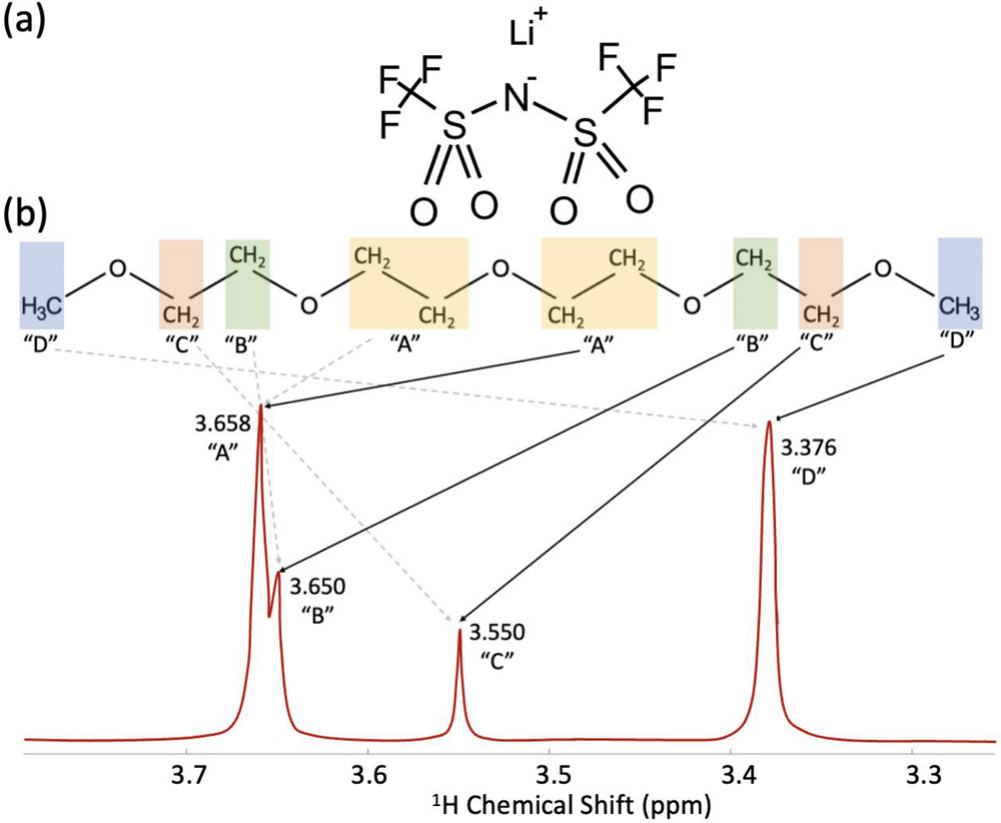
Overall chemical structure of the LiTFSI-G4 electrolyte system: (a) the chemical structure of the LiTFSI salt and (b) the chemical structure of tetraglyme with its corresponding simulated ^1^H NMR spectrum. Color coding differentiates the different proton environments (A–D) ordered by decreasing ^1^H shift, with the arrows indicating the peaks of the corresponding proton environment in tetraglyme. Note that the depicted spectrum has been simulated with arbitrary peak intensity, using the previously reported ^1^H shifts of neat tetraglyme, and does not account for scalar (*J*) coupling effects.

The local solvation structure and dynamics of an electrolyte system, which ultimately dictate functional properties such as conductivity and transference, are defined by the interactions between the salt and the molecular solvent. Specifically, the LiTFSI-G4 electrolyte system is characterized by the solvation interactions of the lithium cation, the TFSI^–^ anion (depicted in Figure 1a), and tetraglyme as the molecular solvent (depicted in Figure 1b). From the NMR perspective, the tetraglyme molecule possesses four distinct proton environments that translate to four different proton chemical shift values in the ^1^H NMR spectra of tetraglyme. Consequently, perturbations of these resonances in the presence of LiTFSI can potentially be used to understand solvation motifs and the corresponding dynamics of the electrolyte system as a function of salt concentration.

Figure 1b depicts the ^1^H NMR spectrum of neat tetraglyme, along with the tetraglyme chemical structure, showing the correspondence between each NMR resonance and the proton environments. For the purposes of this study, the four different proton environments are arbitrarily labeled A–D, from highest to lowest observed chemical shift in the neat solvent; “D” corresponds to the end methyl (CH_3_) groups, and “A” represents the middle methylene (CH_2_) groups (Figure 1b), while “B” and “C” are the CH_2_ groups nearer to the terminating methyl groups. Here, the proton chemical shift values have been taken from the AIST (Advanced Industrial Science and Technology) NMR spectral database, SDBS; the spectrum has been simulated from the reported shifts, and the effects of *J* coupling are ignored.^32^ The chemical shift relies upon the relative amount of shielding and deshielding of the magnetic field experienced by the proton caused by local electronic interactions.^33^ The “A” middle CH_2_ groups are assigned to the highest chemical shift due to proximity to nearby electronegative oxygen atoms, unlike the other groups that lie closer to the end methyl groups without oxygen atoms. In this work, the significance of the NMR spectra relies on an understanding of how cation solvation-induced changes of the local proton environment affect the position of the NMR resonances. Specifically, this study focuses on the relative ^1^H chemical shift values, quantified as the shift difference between observed peaks, rather than changes in the absolute values of the chemical shifts, which may be subject to bulk effects such as magnetic susceptibility that do not report on the local solvation structure.

**Figure 2:**
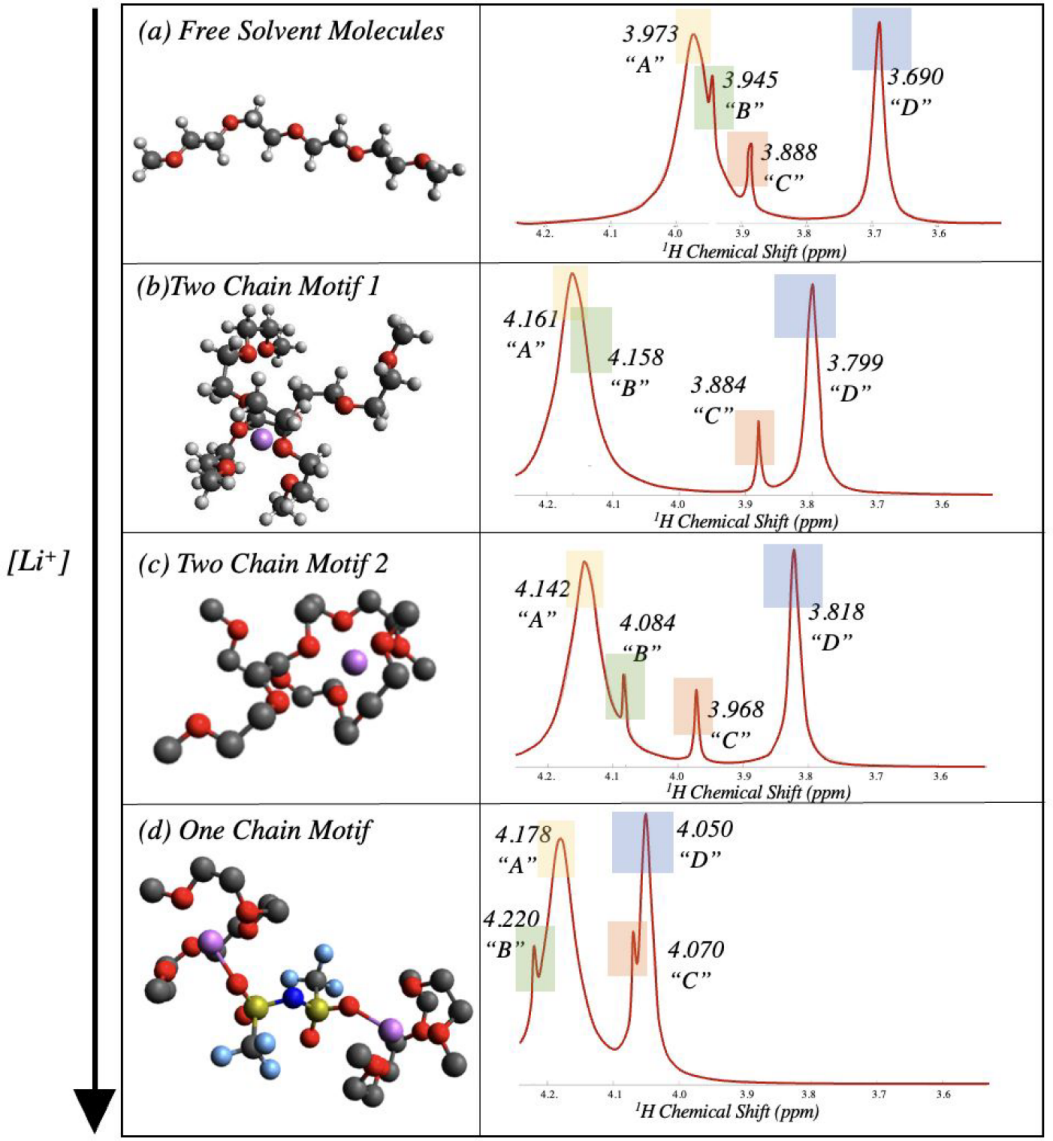
Solvation motifs of the LiTFSI-G4 electrolyte and corresponding simulated ^1^H NMR spectra from DFT-calculated shifts. The left column represents the different solvation structures, identified from MD simulations and further geometry optimized using DFT calculations. The black atoms represent carbon, white atoms represent hydrogen, red atoms are oxygen, purple atoms are lithium, navy atoms are nitrogen, yellow atoms are sulfur, and blue atoms are fluorine. The right column depicts the respective simulated proton spectra, generated based on the DFT-calculated NMR shifts; the spectra are provided for visualization purposes, and scalar (*J*) coupling effects are not included. The relative peak intensity is chosen analogously to that in Figure 1b to ensure peak positions are visible. The simulation was performed using a single averaged peak value for each proton type within the solvent (Supporting Information 1). The peaks corresponding to each type of proton environment are color coded and labeled.

**Figure 3:**
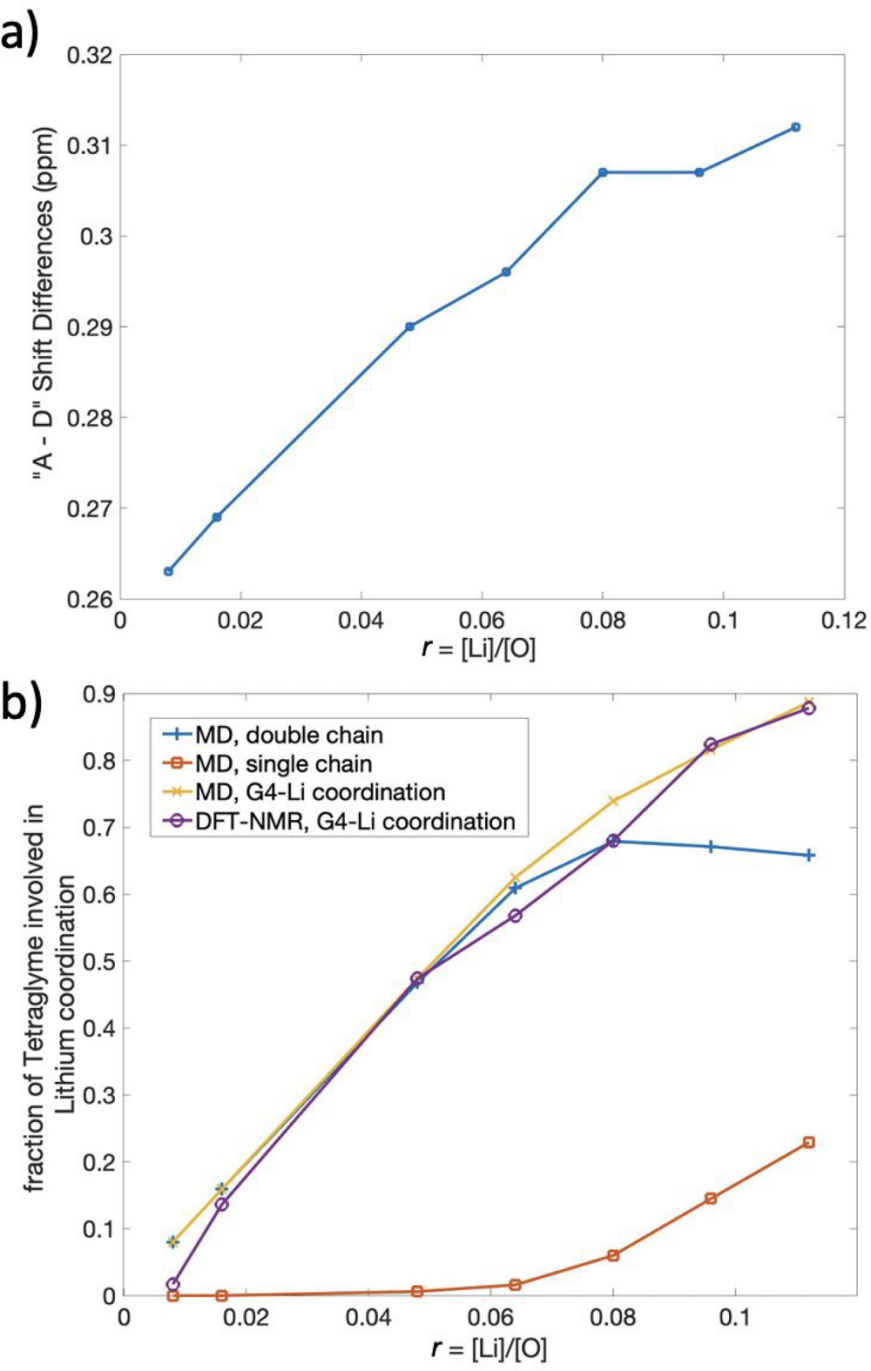
Prediction of fraction of coordinated tetraglyme using MD simulations, ^1^H NMR experiments, and DFT calculations. (a) Experimental ^1^H chemical shift difference between the “A” and “D” resonances of tetraglyme, derived from the ^1^H NMR spectra in Figure S1, as a function of the salt concentration, *r*. (b) Comparison of fraction of coordinated tetraglyme as a function of salt concentration, predicted separately from MD simulations directly, and indirectly from the ^1^H NMR experiments, using DFT-calculated shifts. The MD-simulated data (blue, orange, and yellow traces) reflect the fraction of tetraglyme coordinated in two-chain motifs, one-chain motifs, and the sum of these two fractions. The experimental NMR data (purple trace) reflects conversion of the shift differences shown in (a) to fraction of coordinating tetraglyme, using ^1^H shift values calculated by DFT (see eq 1). The agreement between the total fraction of coordinated tetraglyme (yellow) and the expected values derived from NMR shifts (purple) corroborates the solvation structures and solvation mechanisms of the LiTFSI-G4 system as predicted by MD.

To assess the sensitivity of the NMR spectra to changes in local solvation structure, we first investigated the different solvation structures of the LiTFSI-G4 electrolyte system. Solvation motifs were extracted from MD simulation^16^ and then further geometry-optimized using hybrid functional DFT calculations. In this way, we could understand the evolution of the distinct types of solvation motifs as a function of salt concentration (*r* = [Li^+^]/[O], the ratio between the concentration of lithium cations and that of the oxygen atoms in tetraglyme). Figure 2 presents the three dominant dynamically heterogeneous^34^ motifs evident in the electrolyte system, as determined by analysis of the MD simulations.^16^ As described previously, these motifs have been extracted by considering the local environments of lithium cations in static snapshots of the MD simulations. These motifs correspond to (1) free solvent molecules (Figure 2a), (2) “two-chain” motifs with two solvent molecules and one cation (Figure 2b,c), and (3) “one-chain” motifs with two solvent molecules, two cations, and one anion (Figure 2d). The free solvent motif represents the structure of the tetraglyme molecule when the oxygens of the tetraglyme molecules are not directly coordinated to lithium cations. With increasing salt concentration, the dominant solvation structure shifts to two-chain motifs representing a 2:1 ratio between tetraglyme molecules and lithium cations, with two tetraglyme molecules providing coordinating oxygen sites for one lithium cation. The one-chain motifs become the predominant solvation structure at higher concentrations, which results from the increase in the amount of lithium cations available in comparison to the amount of tetraglyme. These motifs comprise a coordination ratio of 1:1, with one tetraglyme molecule surrounding one lithium cation and the TFSI^–^ anions providing the remaining oxygen sites for coordination (Figure 2d).

The DFT-calculated values predict a change in the difference between the chemical shift values of the “A” and “D” resonances as the dominant solvation structure changes from free solvent molecule to two-chain motifs. The chemical shift difference between the “A” and “D” peaks is calculated as 0.283 ppm for a “free” solvent molecule, whereas this increases to 0.324 or 0.362 ppm for the two-chain motif (depending on the specific structure with an average chemical shift difference of 0.343 ppm). The increase is apparently due to the strong coordination of the lithium cation to the oxygen atoms in the central region of the tetraglyme molecule (i.e., within the “A” region) compared to the end region, according to a chelation-like mechanism. This is readily confirmed by inspection of the MD-derived geometries, in which the Li^+^ resides closer to the centers of both tetraglyme molecules to provide the conformational flexibility for full solvation by six oxygen atoms. Our calculations are consistent with prior experimental and theoretical studies that have reported the tendency of glymes to “wrap around” lithium cations and thereby create a stable, crown-ether-like complex cation.^1^ We note in passing that minor differences may exist between subtypes of two-chain motifs, i.e., those labeled 1 and 2 in Figure 2. Visually, the difference between the two subtypes of two-chain motif reflects subtle conformational changes, which has been observed in past literature comparing gauche/trans conformers of glymes and glyme–cation complexes.^35^ The difference between these two double-chain motifs may be identified, however, through calculated differences in the “C” and “D” chemical shift values, but these are too small to be quantified by experimental measurements. Moreover, these solvation structures may undergo conformation interconversion between motif subtypes on a time scale that is short relative to the NMR measurement (i.e., submillisecond), based on MD results discussed later.^16^ Therefore, we predict that experimental NMR spectra are incapable of distinguishing the two different two-chain motifs, and thus we report the average calculated chemical shift values of these structures.

On the other hand, the predicted spectral difference between free solvent molecules and the one-chain motif is more striking, where in the one-chain motif the “A” and “D” chemical shift difference decreases to 0.128 ppm. This significant decrease appears due to the strong coordination of the Li^+^ cation to every oxygen of the tetraglyme molecule, exhibiting a cyclo-like behavior, and thus a much more uniform proton environment. One unique observation for the single-chain motif is that this is the only structure in which the “A” region does not contain the highest predicted value of the chemical shift; the near-end methylene (CH_2_) groups (“B”) are predicted to possess a slightly larger chemical shift. However, the swap between the relative position of the “A” and “B” resonances is small so as not to be experimentally quantifiable via experimental ^1^H NMR.

To complement computational-based prediction of the importance of the “A” and “D” chemical shift difference, we performed experimental ^1^H NMR measurements of LiTFSI-G4 electrolytes as a function of concentration, paying particular attention to changes in the relative shift difference of the resonances. Figure S1 shows experimental ^1^H spectral data acquired with varying lithium cation concentration. As expected, an increasing difference between the “A” and “D” peaks is observed as the concentration increases (as shown in Figure 3a), aligning with the computational study predicting increasing ^1^H shift differences with increasing two-chain motifs. Moreover, experimental spectra show additional *J* coupling fine structure within the “A” peak as the concentration increases, affirming the diversification of the experimental solvation structures, with different ^1^H NMR chemical shift values, as well as the emergence of two-chain motif #2 (Figure 2), possessing the largest separation between the “C” and “D” chemical shift values.

We used the quantitative experimental difference between “A” and “D” chemical shift values to predict the percentage of the free and coordinated solvation structures in the system using the DFT-calculated shift values. Here we used a simple model equation
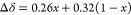
1where Δδ is the experimental shift difference between the “A” and “D” peak and *x* is the fraction of tetraglyme molecules within the free solvent molecule structure, i.e., not undergoing coordination to a lithium cation. Consequently, (1 – *x*) is the fraction of tetraglyme in a two-chain motif structure, where eq 1 is valid for the low-concentration regime. Thus, the presence of the single-chain motif is considered insignificant and is omitted from this simple model. We derive the constant 0.26 ppm from subtracting 0.02 from the DFT-calculated “A” – “D” chemical shift difference for free solvent molecules; analogously, the constant 0.32 ppm is derived from subtracting 0.02 from the DFT-calculated “A” – “D” chemical shift difference for two-chain motifs (averaging the two subtypes). The subtraction of 0.02 ppm accounts for the observed difference in our experimental NMR value of neat tetraglyme and the DFT prediction; this may reflect additional effects such as magnetic susceptibility or temperature-dependent chemical shifts, which were not accounted for in the DFT calculations, or the choice of a limited basis set or functional for reasons of computational time. We argue that this adjustment does not invalidate the use of DFT-calculated shifts because the DFT calculation is equivalent to the chemical shift difference provided in the NMR spectra database (Figure 1b) which indicates that the 0.02 ppm difference is due to external experimental parameters related to our specific NMR measurements.

Just as the combination of ^1^H NMR and DFT provides a predicted fraction of coordinated solvent, the MD simulation results also yield the percentage of tetraglyme solvation motifs as a function of lithium cation concentration in the LiTFSI-G4 system. Comparing solvent versus cation solvation affords further tests of the solvation constructs. Figure 3b depicts the comparison between the fraction of tetraglyme within differing solvation motifs as predicted by MD simulations as well as based on the combination of DFT calculations with experimental NMR data. The calculated fraction of two-chain motifs as predicted by MD simulation (blue trace) increases significantly with salt concentration before leveling off. The fraction of tetraglyme molecules that are coordinated to lithium ions, in both double- and single-chain motifs, increases monotonically (yellow trace). The agreement between these two fractions from the dilute limit to around *r* ≈ 0.08 shows that the solvation structure of the lower-concentration region is dominated by two-chain motifs and free solvent molecules. Consequently, the model equation we have used to understand the lower-concentration regions is corroborated, despite the absence of a term corresponding to the single-chain motif. The absence of single-chain motifs in the lower-concentration region is also supported by the MD data (orange trace), showing the fraction of tetraglyme in this motif is negligible when the lithium cation concentration is small and increases gradually as the concentration increases beyond *r* = 0.08. Promisingly, the DFT/NMR-predicted fraction of two-chain motifs (purple trace) follows an identical trend to the MD data set up until *r* = 0.08. Therefore, we conclude that the MD methodology provides a reliable depiction of the local solvation structure of the LiTFSI-G4 electrolyte system in the low-concentration region (*r* < 0.08). Moreover, we reiterate that the LiTFSI-G4 electrolyte system in the lower-concentration region is dominated by two-chain motifs with a 2:1 ratio between tetraglyme molecule and the lithium ion. The agreement between the DFT-NMR data set and the total fraction of coordinated tetraglyme is striking; this may indicate that the much longer-lived two-chain motif (as discussed later) remains the dominant lifetime-weighted contribution to the appearance of the NMR spectra, despite a global decrease from MD trajectories.

The simple model herein is suitable in the lower-concentration regime yet may deviate at higher concentrations. This static model may be insufficient to predict the fraction of coordinated tetraglyme at higher concentrations using the DFT/NMR methodology because of the increase of the single-chain motifs with increasing concentration. However, this explanation cannot account entirely for the discrepancy, as based on the DFT-calculated shifts (Figure 2), the average chemical shift difference should decrease with increasing fraction of tetraglyme within one-chain motifs, in contrast to the experimental data (Figure 3a). Possibilities for the deviation include the tendency of the MD simulation to overestimate the one-chain motif fraction or the need to account for residence times of the different motifs as discussed below. Future models should consider chemical shift differences weighted by expected lifetimes of the motifs.

**Figure 4:**
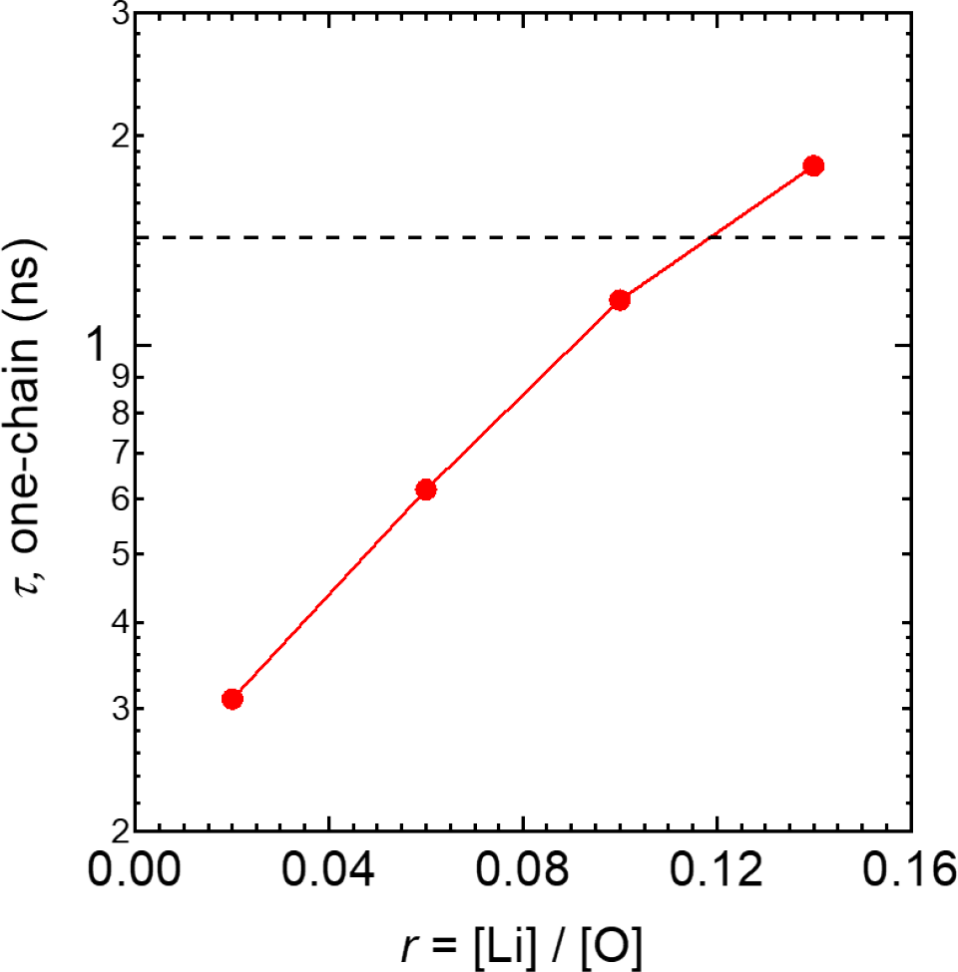
MD-simulated lifetimes of one-chain (ion pair) solvation motifs in LiTFSI-G4 as a function of salt concentration, *r*; the mean residence time of one-chain motif τ is extracted by fitting the stretched exponential function *C*_pair_(*t*) = exp(−(*t*/τ)^β^) to autocorrelation functions obtained from the MD simulations (Figure S2b).^31^ The dashed line indicates the inverse of the Larmor frequency used in the NMR experiments; the intersection with the MD data (red trace) indicates the salt concentration at which a maximum in the ^1^H spin–lattice relaxation rate would be expected (cf. Figure S3).

Finally, we probed the dynamics of the solvation structures in the LiTFSI-G4 system through analysis of residence times in the MD simulations, in combination with experimental NMR relaxometry measurements. For both the two-chain and one-chain motifs, autocorrelation functions were calculated, providing a measure of the time scale of G4 molecules that continuously reside within the same motif. As shown in Figure S2a, typical residence time correlation function of the two-chain motifs are ∼2 μs (at *r* = 0.02), decreasing to ∼0.2 μs (at *r* = 0.14). On the other hand, mean residence times of the one-chain motif are much shorter (Figure S2b), ranging from 0.31 ns (at *r* = 0.02) up to 1.81 ns (at *r* = 0.14), as depicted in Figure 4. Such values are of the right time scale, i.e., similar to the inverse of the ^1^H Larmor frequency in this study (1.4 ns), to provide a contribution to spin–lattice relaxation. Note that while one-chain motif reorganization is not the dominant motional mechanism and primarily depends upon anion dynamics (rather than the longer-lived cation–solvent complex), the presence of a motional process at the Larmor frequency nonetheless provides a mechanism contributing to ^1^H *T*_1_ relaxation of the solvent.^36^ We speculated that the one-chain motif dynamics predicted by MD could therefore be confirmed by ^1^H spin–lattice relaxation measurements.

Figure S3 depicts the ^1^H spin–lattice relaxation rates in tetraglyme for the three ^1^H resonances that could be easily resolved, corresponding to the “A”, “C”, and “D” proton environments (Figure 1). In general, an increase in the relaxation rates is observed with increasing salt concentration, suggesting the relaxation behavior is in the fast motion limit; i.e., the relevant correlation times for motion and/or motif reorganization are shorter than (700 MHz)^−1^ ≈ 1.4 ns. However, a shallow maximum in the *R*_1_ relaxation rate is observed at *r* ≈ 0.12 for the “A” and “C” resonances, implying that at this concentration the motional correlation time is ∼1.4 ns. This value is in excellent agreement with the mean residence time of the one-chain motif at comparable concentration (Figure 4), suggesting that the MD simulations indeed capture the relevant dynamics that dominate *T*_1_ relaxation times. The concurrence between MD and experimental NMR measurements provides confidence in the ability of the present simulation model to provide meaningful information about solvation in concentrated electrolyte systems and will inform future complementary techniques that combine DFT-calculated chemical shifts with dynamic information, going beyond the “static” MD snapshots of tetraglyme fractions considered here.

## Conclusion

To connect the computational-based understanding of LiTFSI-G4 electrolytes with experimental NMR measurements, we have performed complementary MD simulations, DFT calculations, and ^1^H NMR spectroscopy on this system as a function of Li-ion concentration (*r* = [Li^+^]/[O] < 0.12, i.e., less than 2.5 *m* or 2 M). Use of MD simulation pinpoints aspects of the solvation structures and dynamics that could be validated by NMR.

In particular, we obtained MD-derived solvation structures of the tetraglyme solvent molecules as a function of lithium cation concentration.^16^ DFT-calculated ^1^H NMR shifts of these structures showed that the local tetraglyme environment and the corresponding oxygen coordination to the lithium ion strongly affect the ^1^H NMR spectra, specifically the resonances arising from ^1^H in the central region of the solvent molecule that most strongly coordinates with Li^+^. Experimentally, a relative change in the shift of this resonance was observed, supporting findings from DFT calculations. As a quantitative validation of the computational simulations and calculations, we compared MD-simulated results to predicted values using NMR and DFT. Using a simple model employing the DFT-calculated shifts, we converted experimental NMR shifts to fraction of cation-coordinated tetraglyme, which could be compared directly to MD-simulation-based fractions. Agreement between the data sets from these complementary approaches was shown for the low concentration region (*r* < 0.08). The MD simulations suggest the coordination behavior of the tetraglyme solvent molecules closely matches the experimental LiTFSI-G4 system, as quantified by the relative change in ^1^H NMR shift values. Further corroboration is provided by consideration of the dynamics of the solvation structures, comparing MD-derived residence times with ^1^H spin–lattice (*T*_1_) relaxation measurements.

In conclusion, we validate a complementary experimental and computational methodology as a reliable method of understanding solvation mechanisms and dynamics of the LiTFSI-G4 electrolyte system in the moderately concentrated regime. In this low-concentration electrolyte system, two-chain motifs are the dominant solvation structure and comprise the main influence on the ^1^H NMR spectra of tetraglyme. Future work is needed to demonstrate the validity of the methodology in higher concentration regimes, in which more diverse and complicated solvation structures may arise. We further argue that the work opens up the possibility of the use of spectroscopy in extracting information about the probability of certain motifs as well as the corresponding lifetime of each motif.

## Supporting Information Available

DFT-based predictions of ^1^H NMR chemical shifts, selection of functional/basis set combination for the DFT calculations, selection of neat tetraglyme conformers, MD simulation force field parameters, and additional NMR and MD results (PDF)
