# Supplementary material for: Understanding the Solvation Structure of Li-Ion Battery Electrolytes Using DFT-Based Computation and 1H NMR Spectroscopy
Source: J Phys Chem B. 2022 Nov 16;126(47):9893–900. doi: 10.1021/acs.jpcb.2c06415 (PMC9720717; doi:10.1021/acs.jpcb.2c06415)
Supplement: Supplementary file 1 — jp2c06415_si_001.pdf [file jp2c06415_si_001.pdf]

Supporting Information

Understanding the Solvation Structure of Li-ion Battery Electrolytes Using DFT-based Computation and ^{1}H NMR Spectroscopy

Julia Im,[1] David M. Halat,[1,2] Chao Fang,[1,2] Darby T. Hickson,[1,2] Rui Wang,[1,2] Nitash P. Balsara,[1,2] Jeffrey A. Reimer[1,2,*]

[1]*Department of Chemical and Biomolecular Engineering, University of California, Berkeley, Berkeley, California 94720, United States*

[2]*Materials Sciences Division and Joint Center for Energy Storage Research (JCESR), Lawrence Berkeley National Laboratory, Berkeley, California 94720, United States*

* reimer@berkeley.edu

Supporting Information 1 DFT-based predictions of ^{1}H NMR chemical shifts

Gaussian software was used to perform NMR calculations for the different solvation motifs. Single tetraglyme molecules were selected to perform the geometry optimization and NMR calculations for neat tetraglyme. Single lithium ion with two tetraglyme molecules were selected to perform the calculation for the two-chain motifs. Last, single TFSI anion, two lithium ions, and two tetraglyme molecules were selected to perform the calculation for the one-chain motif.

Category corresponds to the characterizations of the proton environment with "D" representing the end methyl groups, "A" representing the middle methylene groups, and "B" and "C" representing the methylene groups between "A" and "D". The proton labelled number represent the auto-generated labels by the Gaussian software when running the NMR calculation.

Table S1. ^{1}H chemical shifts of the individual protons in neat tetraglyme.

Category	Proton Labelled Number	^{1}H Shift (ppm)
D	16	3.9542
D	17	3.5401
D	18	3.5533
C	19	3.7543
C	20	3.817
B	21	4.2007
B	22	3.7651
A	23	4.3224
A	24	3.8384
A	25	4.115
A	26	3.7829

A	27	3.9172
A	28	3.9521
A	29	3.9479
A	30	3.9115
B	31	3.7481
B	32	4.0667
C	33	4.3138
C	34	3.6658
D	35	3.8766
D	36	3.4308
D	37	3.7876

Table S2. ^{1}H chemical shifts of individual protons in double chain motif #1.

Category	Proton Labelled Number	^{1}H Shift (ppm)
D	32	3.8711
D	33	3.7415
D	34	4.204
C	35	3.7381
C	36	4.2827
B	37	4.4792
B	38	3.9291
A	39	4.2849
A	40	3.9351
A	41	3.8993
A	42	4.233
A	43	3.8869
A	44	4.7403
A	45	4.0766

A	46	3.7805
B	47	4.0446
B	48	4.0367
C	49	3.8013
C	50	3.6363
D	51	4.1319
D	52	3.3885
D	53	3.472
D	54	4.0479
D	55	3.4744
D	56	3.5013
C	57	3.981
C	58	3.8007
B	59	4.1158
B	60	4.2397
A	61	4.1033
A	62	4.3702
A	63	3.8331
A	64	4.5511
A	65	4.7922
A	66	3.5003
A	67	3.7973
A	68	4.7983
B	69	4.6091
B	70	3.81
C	71	3.7157
C	72	4.1128
D	73	4.3492
D	74	3.7537
D	75	3.6524

Table S3. ^{1}H chemical shifts of individual protons in double chain motif #2

Category	Proton Labelled Number	^{1}H Shift (ppm)
D	32	3.7681
D	33	4.2084
D	34	3.8559
C	35	3.7197
C	36	4.3123
B	37	4.4435
B	38	3.9395
A	39	4.305
A	40	3.9253
A	41	3.8884
A	42	4.2431
A	43	3.8814
A	44	4.7318
A	45	4.0572
A	46	4.0567
B	47	3.7896
B	48	3.6448
C	49	4.1354
C	50	3.4147
D	51	3.4222
D	52	3.524
D	53	4.0399
D	54	3.4408
D	55	3.9514
D	56	3.8489
C	57	4.0861

C	58	4.2424
B	59	4.1381
B	60	4.2938
A	61	3.8212
A	62	4.6002
A	63	4.0249
A	64	3.8472
A	65	4.8148
A	66	3.4533
A	67	3.7965
A	68	4.8253
B	69	4.6113
B	70	3.8103
C	71	3.7182
C	72	4.1131
D	73	4.3585
D	74	3.7566
D	75	3.6404

Table S4. ^{1}H chemical shifts of individual protons in the single chain motif

Category	Proton Labelled Number	^{1}H Shift (ppm)
D	48	4.6445
D	49	3.7683
D	50	3.7614
C	51	4.2732
C	52	3.6449
B	53	3.7364
B	54	4.697

A	55	3.5373
A	56	4.7619
A	57	4.2751
A	58	3.5823
A	59	3.8743
A	60	4.3549
A	61	4.2474
A	62	3.935
B	63	4.3958
B	64	3.8804
C	65	3.7712
C	66	4.803
D	67	4.2829
D	68	3.8079
D	69	4.2339
D	70	3.6988
D	71	4.6502
D	72	3.8437
C	73	3.6409
C	74	4.1602
B	75	4.8566
B	76	3.7274
A	77	5.593
A	78	3.7124
A	79	3.9269
A	80	3.9411
A	81	4.0032
A	82	4.0895
A	83	5.164
A	84	3.8457
B	85	3.8273

B	86	4.6391
C	87	4.7636
C	88	3.5022
D	89	4.1768
D	90	4.0227
D	91	3.7103

Supporting Information 2 Selection of functional/basis set combination for the DFT calculation

Given the number of calculations and conformations explored in this work, the B3LYP/6-31G(d) functional/basis set combination was optimal for our purpose. To validate the use of the following functional/basis set, we performed DFT calculations using varying functional/basis set on the neat tetraglyme system and compared with the experimental results. Our results show that the usage of B3LYP/6-31G(d) accurately reflects the experimental results and is still time efficient.

Experimental neat tetraglyme NMR spectra based on the NMR spectral database

Proton Classification	Chemical Shift (ppm)
A	3.658
B	3.650
C	3.550
D	3.376

“A-D” Difference in Chemical Shift: 0.282

DFT-based NMR spectra (B3YLY/6-31G(d))

1. Proton Classification	Chemical Shift (ppm)
A	3.973
B	3.945
C	3.888
D	3.690

“A-D” Difference in Chemical Shift: 0.283

Calculation done on varying functional/basis set

Functional	DFT Basis	Calculated energy (A.U.)	A-D Difference in Chemical Shift (ppm)
B3LYP	6-31G(d)	-770.3440	0.283
B3LYP	6-31G(d,p)	-770.3732	0.252
B3LYP	6-311+G(d,p)	-770.6002	0.224
B3LYP	6-311++G(3df,2p)	-770.6360	0.195
B3LYP	Aug-cc-pVTZ	-770.6567	0.196
RHF	6-31G(d)	-765.7161	0.0655
AM1	6-31G(d)	-0.3823*	NMR calculations not implemented in Gaussian
PM3	6-31G(d)	-765.7160	NMR calculations not implemented in Gaussian
MP2	6-31G(d)	-765.7077	0.250
CCSD	6-31G(d)	-765.7079	NMR calculations not implemented in Gaussian
B97D3+	6-31G(d)	-769.8703	0.310
APFD+	6-31G(d)	-769.7249	0.301
wB97XD+	6-31G(d)	-770.1092	0.283

* Note that for AM1, the energies reported represent heats of formation and cannot be compared directly with the other methods.

+ Functionals including dispersion correction

Supporting Information 3 Selection of neat tetraglyme conformers

For our study, the following motif was used for the calculations related to neat tetraglyme molecules. This conformer is generated by geometry-optimizing the tetraglyme molecule from the linear position to its energy minimum.

Conformer 1

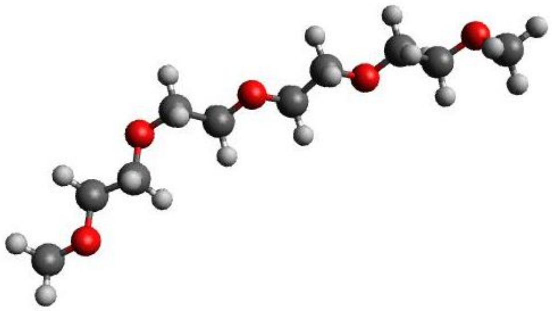

<T T T T> conformation (all oxygen atoms are anti/trans)

Following are conformers 2-4, which are other possible conformers of neat tetraglyme, as derived by MD simulations. These conformers were chosen from random snapshots from MD simulations in which the tetraglyme molecules were not coordinated to Li^+, so they represent common conformers of neat G4. Within each of conformers 2-4, all oxygen atoms are arranged in a gauche manner. The conformers differ in that two dihedral angles (positive or negative) are possible for a gauche configuration; we have explored a representative sample of conformers of this type as revealed by the MD simulations.

Conformer 2

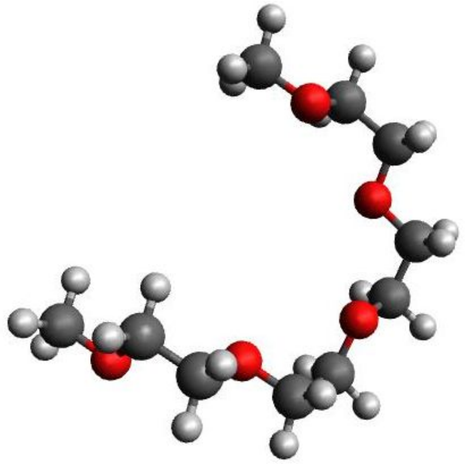

<G G G G> conformation (all oxygen atoms are gauche)

Conformer 3

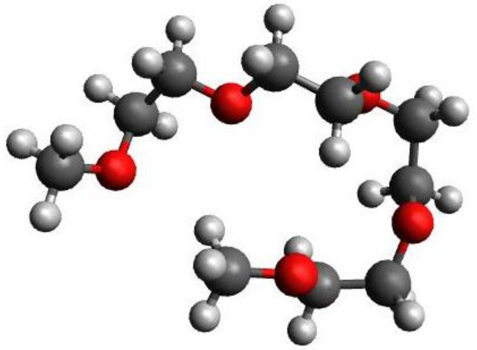

<G G G G> conformation (all oxygen atoms are gauche)

Conformer 4

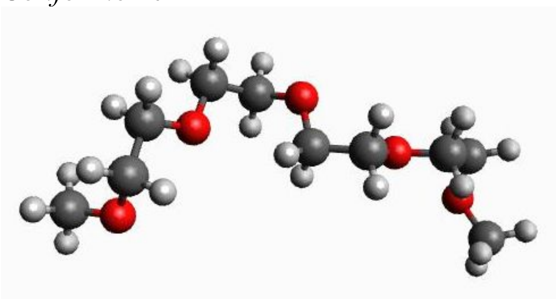

<G G G G> conformation (all oxygen atoms are gauche)

The NMR spectra for each neat tetraglyme conformers were performed and compared based on the "A-D" chemical shift difference. The experimental data of comparison as the "A-D" difference is 0.282 ppm. Conformer 1 strongly agrees with the experimental data. Moreover, the average of the various conformers (average of conformer 1-4, average of conformer 2-4) also resembles the experimental data. Therefore, we can validate the use of conformer 1 as a representative conformer for neat tetraglyme.

Conformer	"A-D" Difference in Chemical Shift (ppm)
1	0.283
2	0.160
3	0.346
4	0.332
2-4 average	0.279
1-4 average	0.281

Supporting Information 4 MD Simulation force field parameters

(1) Lennard-Jones parameters and partial charges

Particles	Particle type	Charge (e)	σ_i (nm)	ϵ_i (kJ/mol)
Lithium cation	Li^+	1.00	0.140	1.67360e+0
Carbon atom ($TFSI^-$)	CT	0.35	0.350	2.76140e-1
Nitrogen atom ($TFSI^-$)	N	-0.66	0.325	7.11280e-1
Oxygen atom ($TFSI^-$)	O	-0.53	0.296	8.78640e-1
Fluorine atom ($TFSI^-$)	F	-0.16	0.295	2.21750e-1
Sulfur atom ($TFSI^-$)	S	1.02	0.355	1.04600e+0
CH3 bead (G4)	CET	0.25	0.375	8.15242e-1
Oxygen atom (G4)	OET	0.50	0.285	4.57534e-1
CH2 bead (G4)	CHA	0.25	0.395	3.82664e-1

(2) Bonded parameters

(a) Bond stretching

$$V_b(r_{ij}) = \frac{1}{2}k_{ij}^b(r_{ij} - b_{ij})^2$$

Bond type	b (nm)	k^b (kJ/mol/nm^2)
CET-CET	1.540000e-1	NA (fixed bond)
CET-OET	1.410000e-1	NA (fixed bond)
OET-CHA	1.410000e-1	NA (fixed bond)
CT-S	1.818000e-1	1.970000e+5

CT-F	1.323000e-1	3.697000e+5
S-N	1.570000e-1	3.113000e+5
S-OT	1.442000e-1	5.331000e+5

(b) Angle vibration

$$V_a(\theta_{ijk}) = \frac{1}{2}k_{ijk}^{a}(\theta_{ijk} - \theta_{ijk}^{0})^2$$

Angle type	k^a (kJ/mol/rad^2)	θ^0 (degree)
CET-CET-OET	4.184352e+2	112
CET-OET-CET	5.024550e+2	112
CET-OET-CHA	4.184352e+2	112
CT-S-N	8.160000e+2	100.2
CT-S-OT	8.700000e+2	102.6
S-CT-F	6.940000e+2	111.8
S-N-S	6.710000e+2	125.6
N-S-OT	7.890000e+2	113.6
F-CT-F	7.810000e+2	107.1
OT-S-OT	9.690000e+2	118.5

(c) Ryckaert-Bellemans proper dihedral

$$V_{rb}(\phi_{ijkl}) = \sum_{n=0}^{5} C_n(cos\,(\phi_{ijkl} - 180^o))^n$$

Dihedral type	$c_0\ (kJ/mol)$	$c_1\ (kJ/mol)$	$c_2\ (kJ/mol)$	$c_3\ (kJ/mol)$	$c_4\ (kJ/mol)$	$c_5\ (kJ/mol)$

CET-CET-OET-CET	7.9532e+0	7.8966e+0	2.7244e+0	-1.8574e+1	0	0
CET-CET-OET-CHA	7.9532e+0	7.8966e+0	2.7244e+0	-1.8574e+1	0	0
OET-CET-CET-OET	3.9514e+0	3.9514e+0	7.9029e+0	0	0	0
CT-S-N-S	4.3690e+0	-2.1179e+1	1.0420e+1	6.3900e+1	0	0
S-N-S-OT	-7.5000e-3	-2.2500e-2	0	3.0000e-2	0	0
F-CT-S-N	6.6100e-1	1.9830e+0	0	-2.6440e+0	0	0
F-CT-S-OT	7.2550e-1	2.1765e+0	0	-2.9020e+0	0	0

Supporting Information 5 Additional NMR and MD results

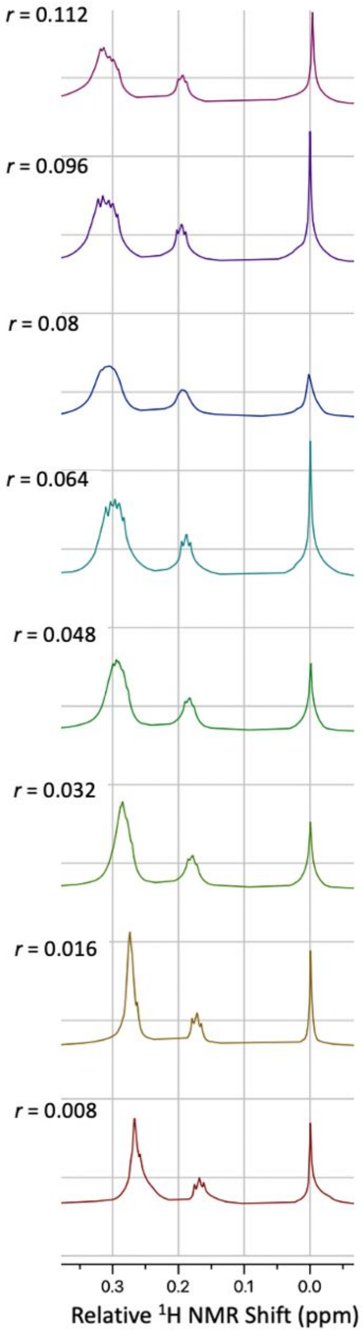

Figure S1. Experimental ^{1}H NMR spectra of the LiTFSI-G4 system as a function of concentration r = [Li^+]/[O]; the shift scale is referenced to the lowest-frequency resonance to highlight the concentration-dependent separation between the resonances. That is, the chain-end "D" resonance (right peak) is referenced to 0 ppm to visualize the overall increasing difference between the "A"

(middle CH_2 proton) and "D" (chain-end CH_3 proton) peaks as a function of salt concentration, *r*. The middle peak corresponds to the "C" (near-end CH_2 protons) resonance in Figure 1, which possesses a much weaker dependence of its chemical shift on the salt concentration. (Note: the position of the "B" resonance overlaps with the more intense signal from "A".)

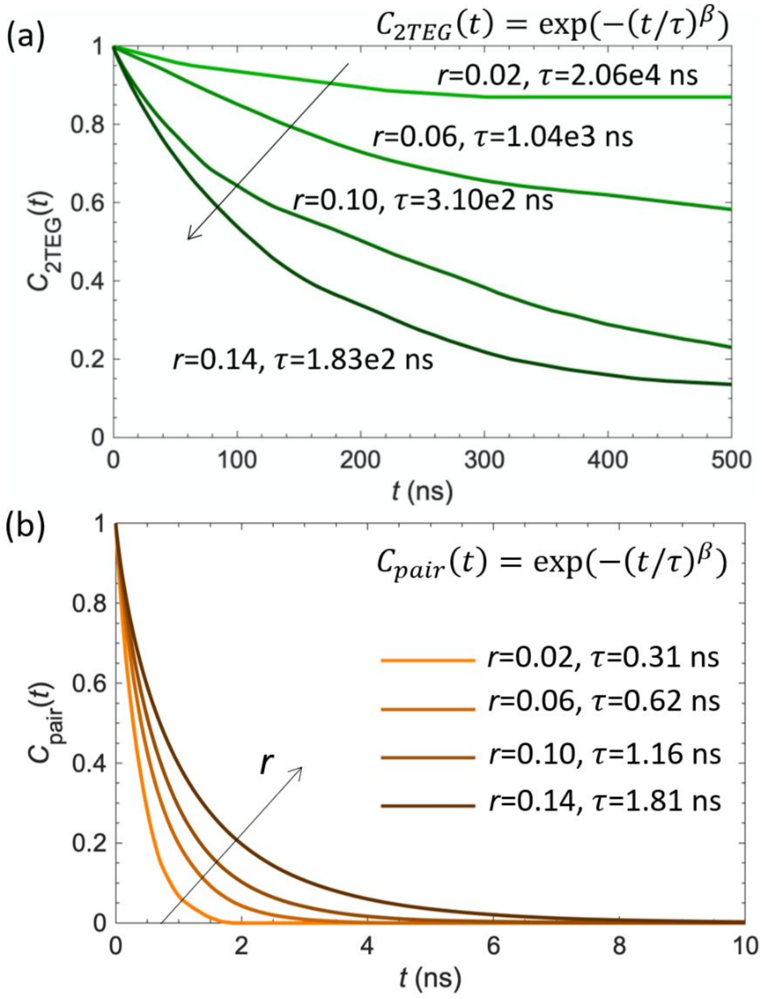

Figure S2. MD-simulated autocorrelation functions for two-chain and one-chain solvation motifs in LiTFSI-G4 as a function of salt concentration *r*; (a) Mean residence time of two-chain motif τ are extracted by fitting the exponential function $C_{2TEG}(t) = \exp\left(-(t/\tau)^{\beta}\right)$, and (b) those for one-chain motif are extracted by fitting the exponential function $C_{pair}(t) = exp\left(-(t/\tau)^{\beta}\right)$.

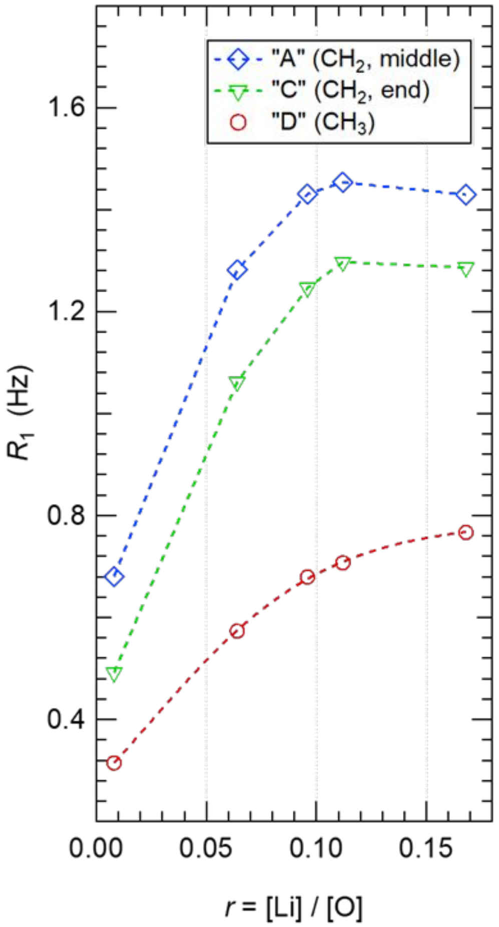

Figure S3. Experimental ^{1}H R_1 spin-lattice relaxation rates for the “A”, “B”, and “D” ^{1}H resonances of tetraglyme as a function of salt concentration, r; a maximum is observed for the former two ^{1}H environments at $r \approx 0.12$.

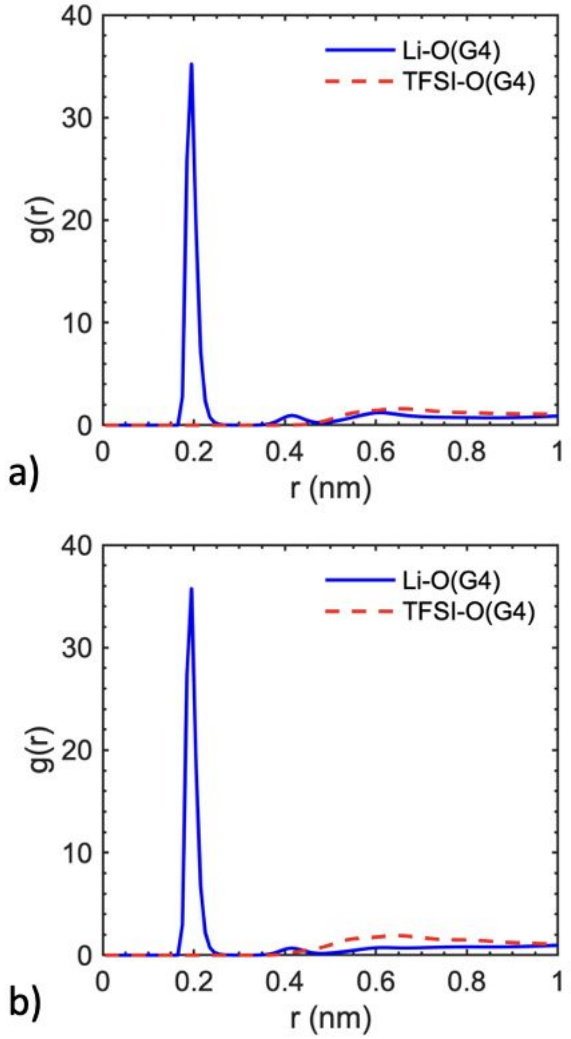

Figure S4. Radial distribution function from MD simulations for LiTFSI-G4 electrolyte system in selected concentrations: (a) $r = 0.048$, and (b) $r = 0.096$. Only long-range interactions between the TFSI - anion and tetraglyme are observed; for tetraglyme molecules, the dominant solvation interaction arises from coordination with Li^+.

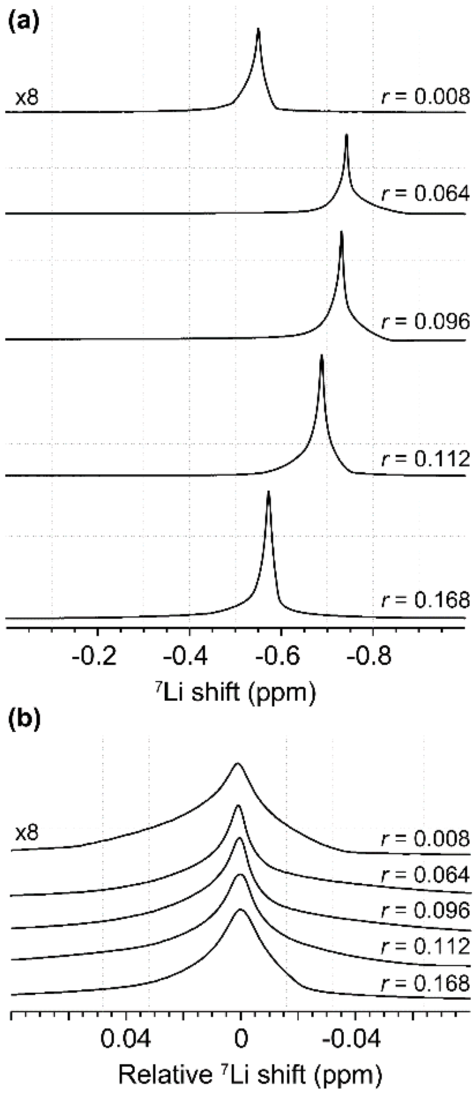

Figure S5. (a) ^{7}Li NMR spectra of selected LiTFSI/tetraglyme electrolytes as a function of concentration. (b) ^{7}Li NMR spectra aligned to peak maximum to compare changes in linewidth. Spectra were acquired at 16.4 T.
